# Targeting Cell Cycle Proteins in Breast Cancer Cells with siRNA by Using Lipid-Substituted Polyethylenimines

**DOI:** 10.3389/fbioe.2015.00014

**Published:** 2015-02-16

**Authors:** Manoj B. Parmar, Hamidreza Montazeri Aliabadi, Parvin Mahdipoor, Cezary Kucharski, Robert Maranchuk, Judith C. Hugh, Hasan Uludağ

**Affiliations:** ^1^Faculty of Pharmacy and Pharmaceutical Sciences, University of Alberta, Edmonton, AB, Canada; ^2^Department of Chemical and Materials Engineering, Faculty of Engineering, University of Alberta, Edmonton, AB, Canada; ^3^School of Pharmacy, Chapman University, Irvine, CA, USA; ^4^Department of Medical Microbiology and Immunology, Faculty of Medicine and Dentistry, University of Alberta, Edmonton, AB, Canada; ^5^Department of Laboratory Medicine and Pathology, Faculty of Medicine and Dentistry, University of Alberta, Edmonton, AB, Canada; ^6^Department of Biomedical Engineering, Faculty of Medicine and Dentistry, University of Alberta, Edmonton, AB, Canada

**Keywords:** breast cancer therapy, cell cycle protein, DsiRNA, CDC20, RAD51, CHEK1, lipid-substituted polymers, non-viral siRNA delivery, Xenograft, siRNA therapy

## Abstract

The cell cycle proteins are key regulators of cell cycle progression whose deregulation is one of the causes of breast cancer. RNA interference (RNAi) is an endogenous mechanism to regulate gene expression and it could serve as the basis of regulating aberrant proteins including cell cycle proteins. Since the delivery of small interfering RNA (siRNA) is a main barrier for implementation of RNAi therapy, we explored the potential of a non-viral delivery system, 2.0 kDa polyethylenimines substituted with linoleic acid and caprylic acid, for this purpose. Using a library of siRNAs against cell cycle proteins, we identified cell division cycle protein 20 (CDC20), a recombinase RAD51, and serine–threonine protein kinase CHEK1 as effective targets for breast cancer therapy, and demonstrated their therapeutic potential in breast cancer MDA-MB-435, MDA-MB-231, and MCF7 cells with respect to another well-studied cell cycle protein, kinesin spindle protein. We also explored the efficacy of dicer-substrate siRNA (DsiRNA) against CDC20, RAD51, and CHEK1, where a particular DsiRNA against CDC20 showed an exceptionally high inhibition of cell growth *in vitro*. There was no apparent effect of silencing selected cell cycle proteins on the potency of the chemotherapy drug doxorubicin. The efficacy of DsiRNA against CDC20 was subsequently assessed in a xenograft model, which indicated a reduced tumor growth as a result of CDC20 DsiRNA therapy. The presented study highlighted specific cell cycle protein targets critical for breast cancer therapy, and provided a polymeric delivery system for their effective down-regulation.

## Introduction

There are significant limitations and side-effects to conventional chemotherapy employed in management of breast cancer. Malignant cells can additionally develop resistance to chemotherapy by changing their molecular (genetic) makeup (Luqmani, 2005; Gillet and Gottesman, 2010). The development of drug resistance in particular warrants a search for alternative and more effective therapies in breast cancer. The treatment of cancer based on RNA interference (RNAi) using small interfering RNA (siRNA) has been a promising approach and it is actively explored as an alternative to chemotherapy (McManus and Sharp, 2002; Kim and Rossi, 2007). Double-stranded synthetic siRNA can be incorporated into the RNA inducing silencing complex (RISC) following the release of the passenger strand and leaving the guide strand of siRNA bound to RISC (Kim and Rossi, 2007; Wilson and Doudna, 2013). The guide strand directs the siRNA–RISC complex to a targeted mRNA. The siRNA–RISC complex can bind and either cleave the target mRNA via endonuclease activity or block the translation of mRNA, resulting in the “silencing” of a specific target (Wilson and Doudna, 2013). However, the binding of siRNA on its own to cell membranes and subsequent uptake is nearly impossible due to its highly anionic nature (Pecot et al., 2011; Bora et al., 2012). The naked siRNA is, moreover, instantly degraded by RNase A in extracellular milieu, resulting in a poor pharmacokinetics profile (Haupenthal et al., 2006). Well-designed carriers are, therefore, necessary to neutralize the anionic charge of siRNA to facilitate its intracellular delivery and inhibit extracellular degradation. We reported a library of cationic polymers based on low molecular weight (2 kDa) polyethylenimine (PEI) that are substituted with different hydrophobic moieties and have shown effective transfection efficiency without significant toxicity (Aliabadi et al., 2011, 2013a,b; Montazeri Aliabadi et al., 2011). Cationic PEI binds to siRNA to provide effective protection against enzymatic degradation, and delivers siRNA into the cells for assembly into the RISC (Aliabadi et al., 2012).

The cell cycle constitutes a series of events that leads to controlled cell division and multiplication (Cooper, 2000). Since deregulation of cell cycle events leads to uncontrolled cell proliferation and is a hallmark of malignancy (Sandhu and Slingerland, 2000; Malumbres and Carnero, 2003), the molecular mediators responsible for abnormal cell cycle regulation are viable targets for siRNA therapy. There are several factors that can deregulate a cell cycle; mutation of a regulatory protein might lead to a loss of binding to its target, or overexpression of a critical protein might lead to constitutive activation of cell cycle. In such cases, malignant cells could proliferate at faster rate than the normal cells, and/or lose the efficiency to detect DNA damage and arrest the progression of cell cycle (Sandhu and Slingerland, 2000; Malumbres and Carnero, 2003). Overexpressed or mutated cell cycle proteins can, therefore, be targeted as the basis of a breast cancer therapy. Many proteins have been found de-regulated during the progression of cell cycle such as the cyclins and cyclin dependent kinases (CDKs) (Vermeulen et al., 2003; Parmar and Uludağ, 2015), and some efforts have been directed to regulate the uncontrolled malignant cell proliferation by delivering siRNA specific to such proteins.

To explore the role of cell cycle proteins as the basis of a breast cancer therapy, this study has undertaken a general approach to identify therapeutically useful targets with polymer-mediated siRNA delivery. Several in-house prepared lipophilic PEIs and a library of siRNAs against cell cycle proteins were screened for this purpose in breast cancer cells. Kinesin spindle protein (KSP), which is required to form a bipolar spindle in mitosis by separating the emerging spindle poles (Blangy et al., 1995; Dagenbach and Endow, 2004), was employed as a reference target since an siRNA against KSP is at early stages of clinical trials (Marra et al., 2013; Tabernero et al., 2013). We hypothesized that silencing critical cell cycle proteins by RNAi would result in reduced cell growth and decreased viability of malignant cells. We further hypothesize that polymeric delivery of siRNA is an effective approach to silence cell cycle proteins in breast cancer cells. The objectives of this study were to find the optimal siRNA delivery system and to identify most effective cell cycle protein target(s) for therapeutic silencing in breast cancer cells. Moreover, we explored the efficacy of siRNA and dicer-substrate siRNA (DsiRNA) *in vitro* and *in vivo*. Unlike the conventional 21-bp double-stranded siRNA, longer DsiRNA can incorporate into the Dicer enzyme in RISC complex, leading to improved silencing efficacies (Amarzguioui and Rossi, 2008).

## Materials and Methods

### Cell-lines

The wild-type (WT) and drug-resistant (R) breast cancer MDA-MB-435 cells were cultured in RPMI 1640 medium, while MDA-MB-231 (WT and R phenotypes) and MCF7 breast cancer cells were cultured in DMEM medium with 10% FBS, 100 U/mL penicillin, and 100 μg/mL streptomycin, and maintained at 37°C and 5% CO_2_. The drug resistance in MDA-MB-435 and MDA-MB-231 cells was developed as described in Aliabadi et al. (2013a,b), and was confirmed periodically by evaluating the IC_50_ (i.e., concentration for 50% cell kill) of doxorubicin in both cell-lines.

### Polymeric carriers and siRNA–polymer complex preparation

Polyethylenimines (2 kDa branched) modified with linoleic acid (LA, 1.65 substitutions/PEI), caprylic acid (CA, 6.0 substitutions/PEI), and α-linoleic acid (αLA, 0.5 substitutions/PEI) were synthesized according to our established protocol (Bahadur et al., 2011; Remant Bahadur and Uludağ, 2011) and the degree of substitution was determined by ^1^H-NMR. The siRNA–polymer complexes were prepared in 150 mM NaCl, and were added to the cells after 30 min of incubation. The siRNA:polymer ratio in the complexes were either 1:2, 1:4, or 1:8 (w/w), and complexes were added to the cells at desired siRNA concentrations (see Figure legends for exact ratios and concentrations). The lipid-based commercial carriers, HiperFect (Qiagen, Valencia, CA, USA), and TurboFect (Thermo Fisher Scientific, Waltham, MA, USA) were included in the screening with the synthesized polymers, and they were used to make complexes as suggested by the manufacturers.

### Cellular uptake of siRNA by flow cytometry and confocal microscopy

To investigate quantitative uptake of siRNA, MDA-MB-435WT were seeded in 24-well plates, and transfected with 6-carboxyfluorescein (FAM)-labeled scrambled siRNA (Cat. No. AM4620; Life Technologies) at 20 and 40 nM concentrations with 1:2, 1:4, and 1:8 siRNA:PEI–LA ratios. Non-labeled scrambled siRNA was used as a negative control. After 24 h of treatment, cells were washed with Hank’s Balanced Salt Solution (HBSS), treated with trypsin/Ethylenediaminetetraacetic acid (EDTA), and the recovered cells were fixed with 3.7% formaldehyde. The uptake of siRNA was quantified using Cell Lab Quanta SC flow cytometer (Beckman Coulter, Brea, CA, USA). The mean fluorescence of the recovered cell population and the percentage of cells showing FAM-fluorescence were determined. Gating of the cell population was such that auto-fluorescence of untreated cells represented 1–2% of the total cell population.

To further investigate qualitative uptake of siRNA, MDA-MB-435WT were grown on glass cover slips (Thermo Fisher Scientific) for 24 h and transfected by non-labeled scrambled siRNA and FAM-labeled scrambled siRNA complexes at 40 nM with 1:2 and 1:8 siRNA to PEI–LA ratios. After 24 h, cells were washed with HBSS, fixed with 3.7% formaldehyde and mounted on a slide using in-house prepared mounting medium (poly vinyl alcohol in glycerol) with 4′,6-diamidino-2-phenylindole (DAPI, Life Technologies) to stain nuclei and wheat germ agglutinin, tetramethylrhodamine conjugate (Invitrogen) to stain cytoplasmic membrane. Prepared slides were studied using 40 × 1.3 oil plan-Apochromat lens in Laser Scanning Confocal Microscope (LSM710, Carl Zeiss AG, Oberkochen, Germany), and using ZEN 2011 software. The number of siRNA–polymer complexes per cell was determined by Imaris software (Bitplane, Belfast, UK). The similar uptake study was performed using MCF7 cells (Figure S1 in Supplementary Material).

### Screening of cell cycle proteins

The human siGENOME siRNA Library on Cell Cycle Regulation (Dharmacon, Lafayette, CO, USA) was used to screen 169 siRNAs to determine the potential cell cycle proteins that can be used as siRNA targets in breast cancer cells. The siRNAs were formulated as a mixture of four different sequences in the library targeting specific protein at four different sites. MDA-MB-435R and MDA-MB-231R cells were cultured in 96-well plate and transfected with 54 nM of each cell cycle protein siRNA at 1:4 siRNA:PEI–LA ratio. In order to assess the sensitizing effect of siRNA for a drug, doxorubicin (Sigma-Aldrich) was added after 48 h of siRNA treatment at 5 μg/mL concentration. The MTT [3-(4 5-dimethylthiazol-2-yl)-2 5-diphenyltetrazolium] assay was performed after 72 h of treatment. The MTT (Sigma-Aldrich, St. Louis, MO, USA) was added to the cells at 1 mg/mL final concentration in HBSS and the cells were incubated for 45 min at 37°C and 5% CO_2_. Soluble MTT is transformed into insoluble formazan crystals during this time by the activity of mitochondrial dehydrogenase enzymes, giving a measure of cellular activity (Sumantran, 2011). Dimethyl sulfoxide (DMSO) was added to the well to dissolve the crystals of MTT dye and the optical density was measured at 570 nm. The percentage viability was calculated as follows: 100% × (absorbance of cells treated with an siRNA complex/absorbance of untreated cells), where the activity of untreated cells was taken as 100% cell growth.

### Validation of identified targets and combinational siRNA therapy

The CDC20, RAD51, and CHEK1 were identified as potential targets based on an initial screening of siRNAs against cell cycle proteins. For validation, individual siRNAs against these cell cycle proteins were obtained from Dharmacon (CDC20: Cat. No. D-003225-10; RAD51: Cat. No. D-003530-02, and; Cat. No. CHEK1: D-003255-06) and delivered to cells at 20 and 40 nM of siRNA concentrations and 1:2, 1:4, and 1:8 siRNA:carrier ratios (in triplicate). Another well-studied cell cycle protein, KSP (siRNA against KSP: Cat. No. AM16706, Life Technologies) and a scrambled siRNA (Cat. No. AM4635, Life Technologies) was included in the validation study. The siRNAs were evaluated in MDA-MB-435, MDA-MB-231, and MCF7 cells by using PEI–LA and PEI–CA as indicated in Figure legends (Figures S2 and S3 in Supplementary Material). The combinational siRNA therapy was performed using 20 nM of each CDC20, RAD51, CHEK1, KSP, and scrambled siRNA with the final siRNA concentration of 40 nM. The siRNA to PEI–LA ratio was 1:2 in MDA-MB-435WT and 1:8 in MDA-MB-435R cells at 40 nM of combinational siRNA. The MTT assay was performed after 72 h of combinational treatment as indicated above. The sensitizing effect of siRNA for doxorubicin was determined using MDA-MB-435R cells at 20, 40, and 60 nM concentrations of siRNA with 1:2, 1:4, and 1:8 siRNA: PEI–LA ratios. Doxorubicin (5 μg/mL) was added to cells after 48 h of treatment with siRNAs and inhibition of cell growth was assessed by the MTT after 72 h of incubation.

### Quantification of transcripts by droplet digital PCR

The MDA-MB-435WT were seeded in six-well plates, and transfected with siRNA complexes at 40 nM (1:2 siRNA:PEI–LA ratios). Total RNA was isolated from MDA-MB-435WT after 24 and 48 h of treatment using TRIzol (Invitrogen). Two microgram of total RNA was converted into cDNA using M-MLV reverse transcriptase (Invitrogen) according to manufacturer’s instruction. The droplet digital PCR (ddPCR) was performed using 10 ng of each cDNA sample and ddPCR supermix for probes by employing QX100 ddPCR system (Bio-Rad, Hercules, CA, USA). The PrimeTime qPCR assays for CDC20 (Assay ID, Hs.PT.58.41063796), RAD51 (Assay ID, Hs.PT.58.38809475), and CHEK1 (Assay ID, Hs.PT.58.3518318) were ordered from IDT (Coralville, IA, USA), while TaqMan gene expression assays for KSP (Assay ID, Hs00189698_m1) and a reference gene, β-actin (Assay ID, Hs01060665_g1) were purchased from Life Technologies. The ddPCR conditions comprised of an initial denaturation for 10 min at 95°C followed by 45 cycles of denaturation for 30 s at 94°C and annealing for 1 min at 60°C, and the final extension for 10 min at 98°C. Template DNA was omitted from the ddPCR reaction as a no template control (NTC) and the results of ddPCR were analyzed using the QuantaSoft Software (Bio-Rad). The absolute copy number of targeted gene was divided by the absolute copy number of a reference gene β-actin and presented as percentage based on untreated cells (100%).

### Targeting cell cycle proteins with DsiRNA

The DsiRNA, having displayed superior efficacy (i.e., sub-nM concentrations) in previous studies (Snead et al., 2013), was also explored to confirm the validity of chosen targets and improve therapeutic efficacy with our carriers. Three DsiRNAs targeting different locations of the mRNA transcript for CDC20, RAD51, and CHEK1 were obtained from IDT, namely CDC20-1 (Cat. No. HSC.RNAi.N001255.12.1), CDC20-2 (Cat. No. HSC.RNAi.N001255.12.2), CDC20-3 (Cat. No. HSC.RNAi.N001255.12.3), RAD51-1 (Cat. No. HSC.RNAi.N001164269.12.1), RAD51-2 (Cat. No. HSC.RNAi.N001164269.12.2), RAD51-3 (Cat. No. HSC.RNAi.N001164269.12.3), CHEK1-1 (Cat. No. HSC.RNAi.N001114121.12.1), CHEK1-2 (Cat. No. HSC.RNAi.N001114121.12.2), and CHEK1-3 (Cat. No. HSC.RNAi.N001114121.12.3) with scrambled DsiRNA (Cat. No. DS NC1). The MDA-MB-435WT and MDA-MB-435R cells were transfected with 20 and 40 nM of DsiRNAs at 1:2, 1:4, and 1:8 DsiRNA:PEI–LA ratios. The sensitizing effect of DsiRNAs for doxorubicin was determined in MDA-MB-435R with the same DsiRNA concentrations by adding doxorubicin (5 μg/mL) after 48 h of DsiRNA treatment. The MTT assay was performed after 72 h of DsiRNA treatment (24 h of doxorubicin treatment). The inhibition of cell growth by DsiRNAs against these cell cycle protein targets were additionally determined in MDA-MB-231WT and MCF7 cells at 40 and 60 nM of DsiRNA using various ratios of DsiRNA:PEI–LA (Figure S4 in Supplementary Material).

### Animal study

All experimental protocols using animals were approved by the Animal Care and Use Committee: Health Sciences at the University of Alberta in accordance with the directions of the Canadian Council on Animal Care. The athymic NCRNU nude mice (4–6 weeks old male) to be used as a xenograft model were obtained from Taconic Biosciences, Inc. (Hudson, NY, USA). Approximately three million MDA-MB-435WT cells were injected subcutaneously into the right flank of the mice, and tumor growth was monitored every 3 days. When the tumor volume reached 60–100 mm^3^ [measured by external calibers as indicated in Aliabadi et al. (2013b)], mice were put on the study by injecting DsiRNA and polymer complexes subcutaneously in the vicinity of the tumors. The mice were treated by 2 μg of DsiRNA/mouse (scrambled or CDC20-1) with 1:8 DsiRNA:PEI–LA ratio. All mice were divided into two groups. First group of mice received weekly injections of scrambled DsiRNA and CDC20-1 DsiRNA (*n* = 7 in each study group) for 3 weeks. The second group of mice received the same siRNA treatment, but at bi-weekly injection intervals (*n* = 7 in each study group). The subsequent tumor volumes were measured twice a week with 3- and 4-day intervals. Any mouse with large tumor volume (>1500 mm^3^), necrotic spot on tumor, or excessive (>20%) weight loss was euthanized before the end-time point of the study for humane considerations and excluded from the entire study. Relative tumor volumes were calculated by normalizing the tumor volumes at different time points with the initial tumor volume (i.e., at the time a mouse is included in the study; taken at 100% initially).

### Statistical analysis

All results were presented as mean ± SD and analyzed by unpaired Student’s *t*-test. The significance (*p* < 0.05) was typically determined by comparing specific siRNA-treated groups to that of scrambled siRNA-treated groups. The significantly different treatment groups are indicated with an asterisk (*) in Figures, with reference groups indicated in the Legend.

## Results

### Initial screening of carriers

**Figure 1:**
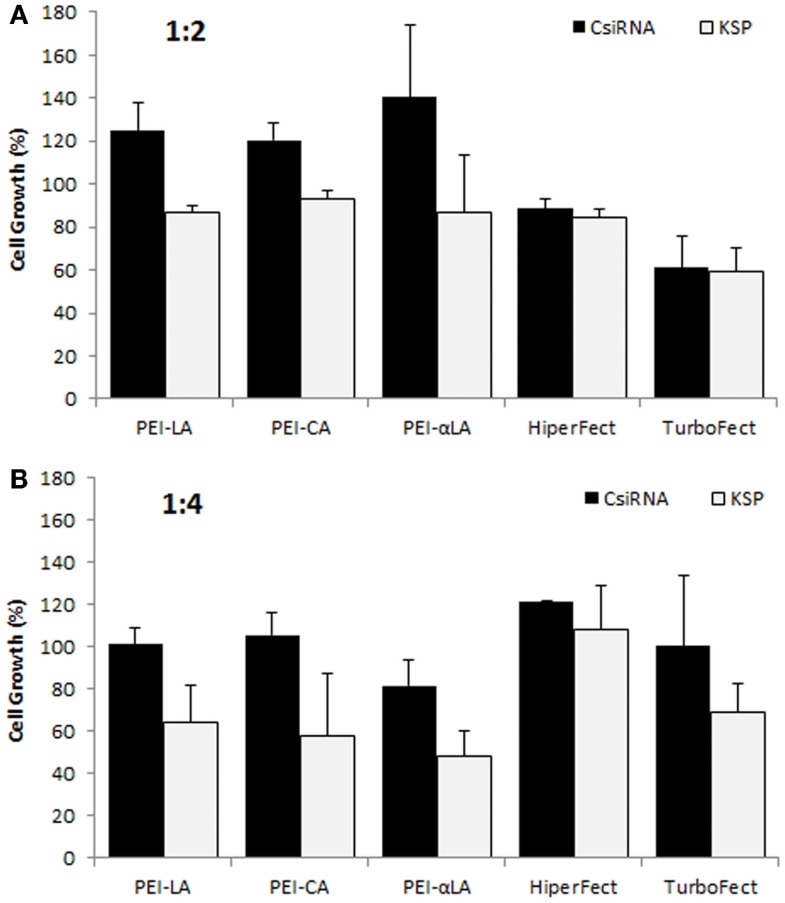
**Screening of polymeric carriers in MDA-MB-435WT cells at 1:2 (A) and 1:4 (B) siRNA to carrier ratios with 20 nM of scrambled siRNA (control; CsiRNA) and KSP siRNA**. Polyethylenimines (PEI) substituted with linoleic acid (LA), caprylic acid (CA), and α-linoleic acid (αLA) were used in the initial screen along with two commercially available carriers.

We previously reported successful delivery of siRNA for specific targets in breast cancer cells using low molecular weight PEI-based polymers (Aliabadi et al., 2011, 2013a,b; Montazeri Aliabadi et al., 2011). As the efficacy of polymers and designed siRNAs was different for each targeted protein, we screened several polymers to identify effective carrier for cell cycle proteins. We screened the PEIs substituted with LA, CA, and αLA and used siRNA against KSP in MDA-MB-435WT cells for this purpose. All chosen polymers were effective for the delivery of KSP siRNA at 1:2 and 1:4 siRNA:polymer ratios employed (Figure 1). The effectiveness of commercial carriers HiperFect and TurboFect was not evident under similar conditions. Among the polymers, PEI–LA was chosen for further studies since: (i) this polymer was equivalent in potency to other polymers, and (ii) it was previously used with other targets and in animal models with success (Aliabadi et al., 2013b).

### Cellular uptake of siRNA

**Figure 2:**
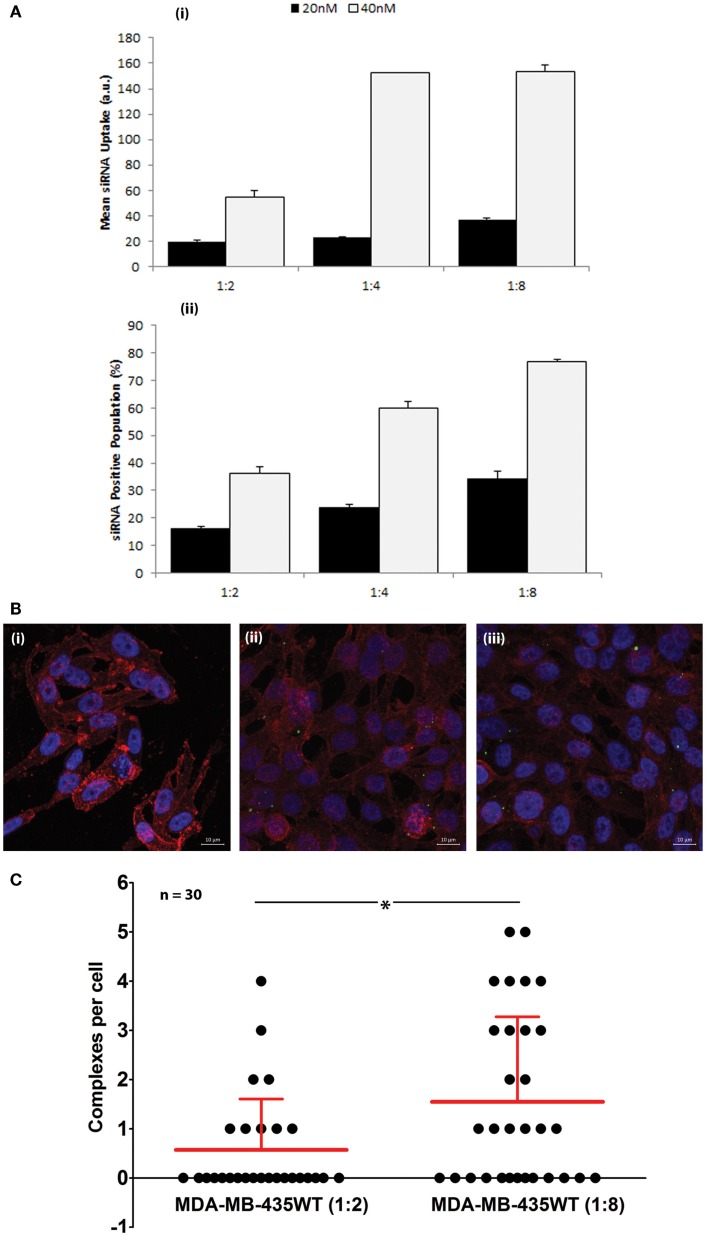
**(A)** Uptake of FAM-labeled siRNA complexes at 20 and 40 nM siRNA using 1:2, 1:4, and 1:8 siRNA:PEI–LA ratios. The MDA-MB-435WT cells were incubated with the complexes for 24 h and recovered for flow cytometry analysis. The results were summarized as mean FAM-siRNA per cell (top) and FAM-siRNA positive cell population (bottom). **(B)** Confocal microscopy to determine the uptake of FAM-labeled siRNA complexes at 40 nM siRNA with 1:2 (ii) and 1:8 (iii) siRNA:PEI–LA ratios after 24 h treatment. Purple, red, and green colors represent nuclei, cytoplasm, and siRNA complexes, respectively. Non-labeled scrambled siRNA was transfected as a control in MDA-MB-435WT (i). **(C)** The number of visible complexes per cell (as quantitated from confocal microscopy images) at 1:2 and 1:8 siRNA:PEI–LA ratios. The uptake was significantly different between 1:2 and 1:8 ratios (*p < 0.05).

The cellular uptake of FAM-labeled scrambled siRNA was performed by flow cytometry to determine the efficiency of PEI–LA to deliver siRNA in MDA-MB-435WT cells (Figure 2A). An equivalent mean fluorescence and FAM-positive cell population was observed between non-treated cell and cells exposed to non-labeled siRNA (data not shown), indicating no cellular auto-fluorescence as a result of siRNA delivery. The siRNA uptake (mean fluorescence) and FAM-labeled siRNA positive cells were dependent on siRNA:PEI–LA ratios, and they were both higher at 40 nM siRNA as compared to 20 nM siRNA concentration, as expected (Figure 2A). A significant difference was found in FAM-labeled siRNA positive cells between 1:4 and 1:8 ratios at 40 nM despite a similar mean fluorescence, suggesting that a higher fraction of MDA-MB-435WT cells were transfected at 1:8 ratio. In order to quantify the number of siRNA–polymer complexes per cell, confocal microscopy was employed and the number of particles was calculated in each cell after taking image by z-stacking (Figure 2B). Non-labeled siRNA was delivered in MDA-MB-435WT (Figure 2Bi) as a control to observe any auto-fluorescent particles; no fluorescent particles were found, which confirmed a lack of auto-fluorescence in confocal microscopy as well. The amount of siRNA–polymer complexes per cell was significantly different between 1:2 (Figure 2Bii) and 1:8 (Figure 2Biii) siRNA:polymer ratios (Figure 2C). Both flow cytometry and confocal microscopy indicated a better delivery by PEI–LA at higher ratio of siRNA:PEI–LA. A similar uptake study using flow cytometry and confocal microscopy was additionally performed with MCF7 cells (Figure S1 in Supplementary Material) and the results again indicated a better delivery with a higher siRNA:PEI-LA ratio.

### Screening of cell cycle proteins

**Figure 3:**
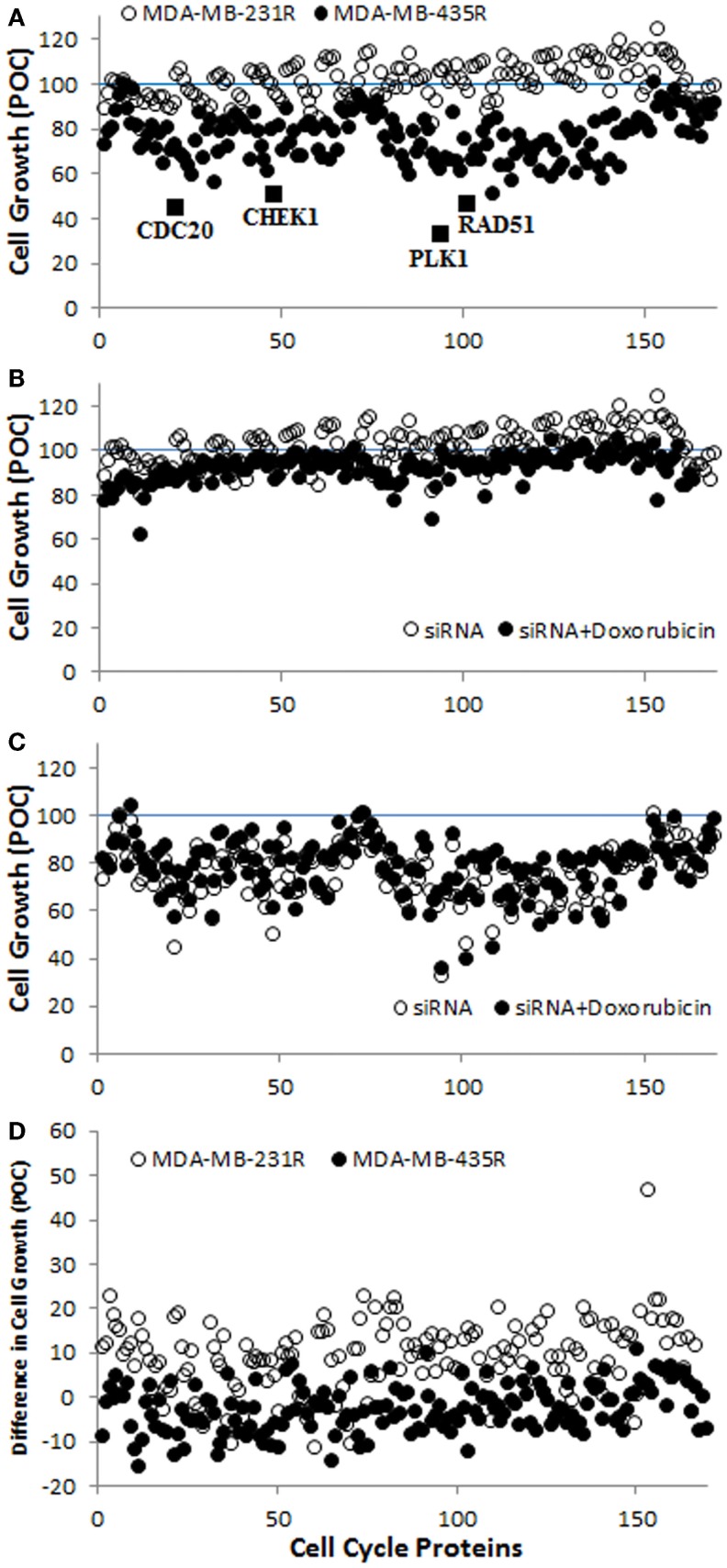
**(A)** Screening of the library of siRNAs against cell cycle proteins using MDA-MB-231R and MDA-MB-435R cells. The identified cell cycle proteins, CDC20, RAD51, and CHEK1 are indicated with the positive control, PLK1. A specific siRNA against PLK1 (Polo-like kinase 1) was provided with the cell cycle proteins; it was meant to assure the functioning of siRNA delivery and was not pursued in this study. **(B,C)** The results of siRNA treatment alone and siRNA/doxorubicin combinational treatment in MDA-MB-231R **(B)** and MDA-MB-435R **(C)**. **(D)** The difference in cell growth between siRNA treatment alone and the combinational treatment. Inhibition of cell growth in all cases was expressed as a percentage of control (POC), which was calculated as a percentage of cell growth (from MTT Assay) for scrambled siRNA-treated cells.

A screening of 169 siRNAs against cell cycle proteins was performed using PEI–LA for delivery. The outcome was based on inhibition of cell growth as assessed by the MTT assay (Figure 3A). The growth inhibition was more significant in MDA-MB-435R cells (up to >50% of control) compared to MDA-MB-231R cells (typically <25% of control). Based on the response of MDA-MB-435R cells, cell division cycle protein 20 (CDC20), the recombinase RAD51, and the serine–threonine protein kinase CHEK1 were identified as potential targets as >50% inhibition of cell growth was achieved by the treatment of siRNA alone for these targets (Figure 3A). In a subsequent study, doxorubicin was added to the cells after 48 h of siRNA addition to determine whether siRNA treatment resulted in sensitizing the cells to doxorubicin. The cell growth of MDA-MB-231R was >70% after dual therapy of siRNA and doxorubicin, except silencing of cyclin D3 (62%; Figure 3B). The MDA-MB-435R cells were more responsive to siRNA therapy, but the sensitizing effect of doxorubicin was not immediately clear in these cells (Figure 3C). The difference in cell growth between siRNA treatment alone and the dual therapy of siRNA/doxorubicin is presented in Figure 3D. The difference in cell growth was 10–25% for many cell cycle proteins in MDA-MB-231R, except homeodomain interacting protein kinase 2 (HIPK2, 46%), which indicates a sensitizing effect for doxorubicin. However, the cell growth of MDA-MB-231R after siRNA/doxorubicin therapy was again >75% (Figure 3B), which was equivalent to siRNA alone.

### Validation and further evaluation of identified targets

**Figure 4:**
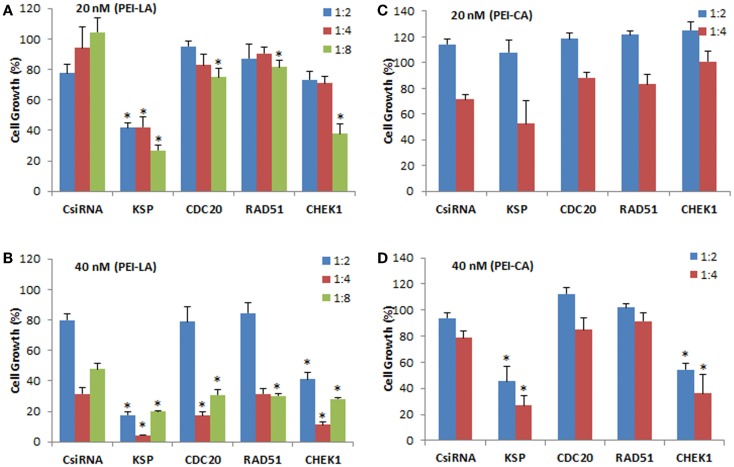
**Validation of KSP, CDC20, RAD51, and CHEK1 in MDA-MB-435WT cells at 20 nM (A,C) and 40 nM (B,D) siRNA concentrations**. The results from PEI–LA were summarized in **(A,B)** (siRNA:polymer ratios of 1:2, 1:4, and 1:8) while the results from PEI–CA were summarized in **(C,D)** (siRNA:polymer ratios of 1:2 and 1:4). Scrambled siRNA (CsiRNA) was used as a control. Significant (*p < 0.05) decreases in the cell growth were observed in specific siRNA-treated group compared to CsiRNA.

The individually prepared siRNAs against the selected cell cycle proteins were used to determine the effectiveness of siRNA therapy. Different siRNA concentrations and siRNA:PEI–LA ratios were explored for this purpose. The inhibition of cell growth was 77 and 62% by delivering KSP siRNA to MDA-MB-435WT cells at 20 nM (1:8) and 40 nM (1:2) siRNA compared to scrambled siRNA using PEI-LA, respectively (Figures 4A,B). However, 1:4 and 1:8 siRNA:polymer ratios at 40 nM siRNA were found more toxic based on the inhibition of cell growth of scrambled siRNA (Figure 4B). Significant decrease in cell growth was observed with 20 and 40 nM CHEK1 siRNA at 1:8 and 1:2 ratios compared to scrambled siRNA, respectively, while cell growth was not significantly decreased with CDC20 and RAD51 siRNA in MDA-MB-435WT cells (Figures 4A,B). This study was repeated with PEI–CA as the delivery agent and the obtained results were similar (Figures 4C,D); a significant inhibition of cell growth was found upon KSP and CHEK1 siRNA treatment at 40 nM siRNA concentration. Consistent with the library screens, the siRNA treatment was not effective in MDA-MB-231WT and MDA-MB-231R cells at 54 nM siRNA using PEI–LA and PEI–CA at 1:2, 1:4, and 1:8 siRNA:polymer ratios (Figures S2 and S3 in Supplementary Material). Similarly, inhibition of cell growth was not significant by delivering these siRNAs to MCF7 cells using PEI-LA (40 and 60 nM siRNA) and PEI–CA (20 and 40 nM siRNA) at 1:2, 1:4, and 1:8 siRNA:polymer ratios (Figures S2 and S3 in Supplementary Material).

**Figure 5:**
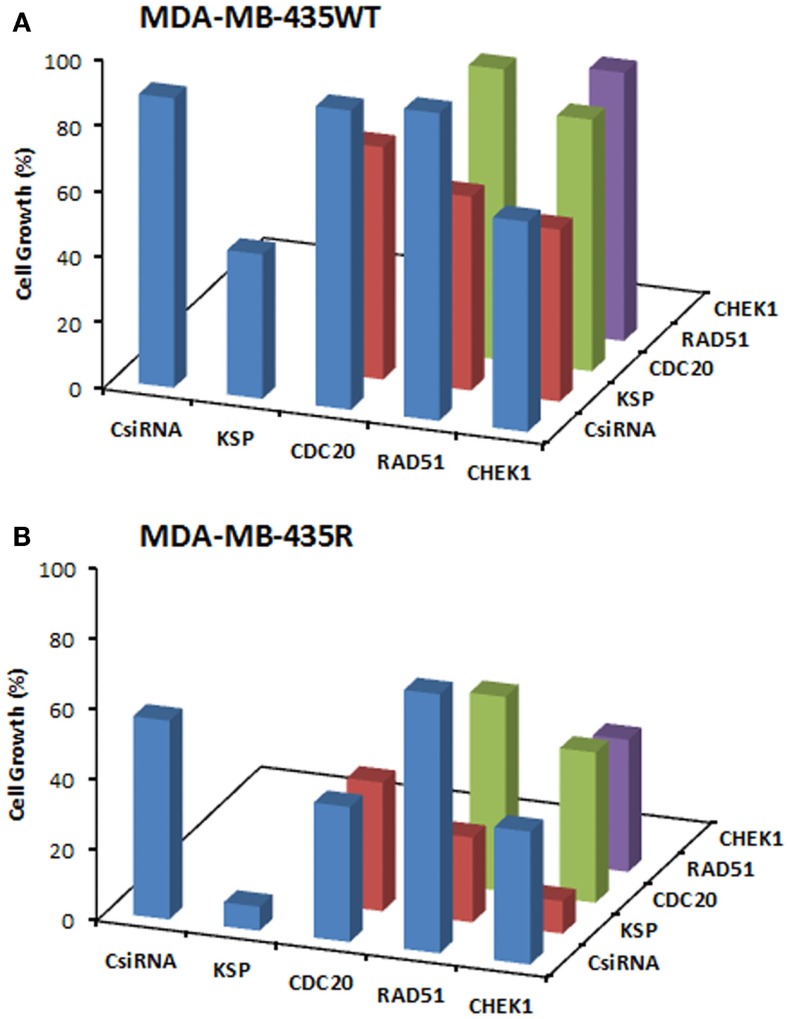
**Combinations of siRNA (20 + 20 nM) treatments among KSP, CDC20, RAD51, and CHEK1 siRNA in MDA-MB-435WT (A) with siRNA:PEI-LA ratio of 1:2 and MDA-MB-435R (B) with siRNA:PEI–LA ratio of 1:8**. Scrambled siRNA (CsiRNA) was used as a control on its own as well as in combination with other siRNAs. The SD (not shown) was <5% for all groups.

We next explored dual delivery of siRNAs with the expectation that if two essential cell cycle proteins are down-regulated simultaneously, cell cycle could be disrupted more significantly with a more pronounced treatment effect. The combinational siRNA therapy was performed using MDA-MB-435WT and MDA-MB-435R cells with 40 nM total siRNA concentration (Figure 5). KSP siRNA, on its own, was highly effective to achieve significant cell death compared to scrambled siRNA. However, cell growth was not drastically decreased using KSP siRNA when co-delivered with CDC20, RAD51, and CHEK1 siRNAs compared to KSP siRNA delivery alone. Similar results were observed with the combinations of CDC20, RAD51, and CHEK1 siRNAs; (i) combining CDC20 with KSP siRNA did not lead to any more inhibition of cell growth with either siRNAs alone, (ii) a combination of RAD51 and CDC20 siRNAs led to a greater inhibition of cell growth than RAD51 siRNA alone, but not CDC20 siRNA alone, and (iii) CHEK1 siRNA combinations with RAD51 and CDC20 siRNA did not lead to greater inhibition of cell growth than CHEK1 siRNA alone. Taking together, these results indicated no synergistic effect with combinational siRNA therapy.

**Figure 6:**
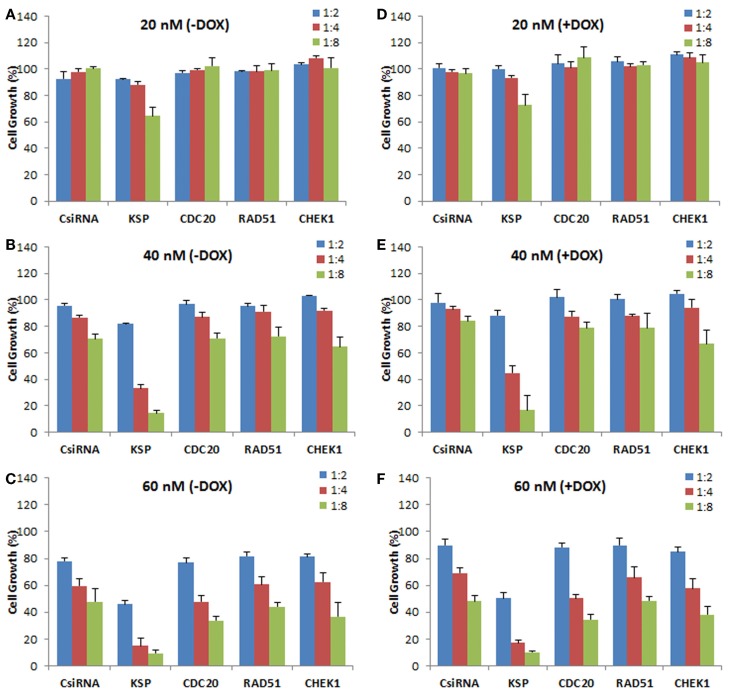
**Effect of siRNA on doxorubicin cytotoxicity in MDA-MB-435R cells**. The cells were first treated with siRNA at 20 nM **(A,D)**, 40 nM **(B,E)**, and 60 nM **(C,F)** with 1:2, 1:4, and 1:8 siRNA:PEI-LA ratios, followed by treatment with buffer [**(A–C)**; −DOX] or doxorubicin [**(D–F)**; +DOX]. Scrambled siRNA (CsiRNA) was used as a control.

We next explored the effect of siRNA delivery on the doxorubicin response of the cells, with the purpose of assessing whether silencing the chosen targets could sensitize the cells to doxorubicin treatment (i.e., further inhibit cell growth as compared to doxorubicin treatment alone). A range of siRNA doses was employed (20, 40, and 60 nM) as well as siRNA:carrier ratios (1:2, 1:4, and 1:8) for a full silencing effect. The MDA-MB-435R cells were subsequently exposed to doxorubicin after 48 h of siRNA treatment targeting the cell cycle proteins. No significant effect of the siRNA treatment was observed at 20 nM siRNA, resulting in no sensitizing effect of doxorubicin in MDA-MB-435R cells (Figures 6A,D). KSP siRNA was the most effective siRNA at 40 nM with 1:8 siRNA:polymer ratio compared to CDC20, RAD51, and CHEK1 siRNAs (Figure 6B). However, the sensitizing effect on doxorubicin was again not observed in the combinational therapy of siRNA (40 nM) and doxorubicin (Figure 6E). Similarly, no sensitizing effect of doxorubicin was observed at 60 nM siRNA concentrations with the drug treatment compared to no drug treated group at the same siRNA concentration (Figures 6C,F).

### Down-regulation of targeted protein transcripts

**Figure 7:**
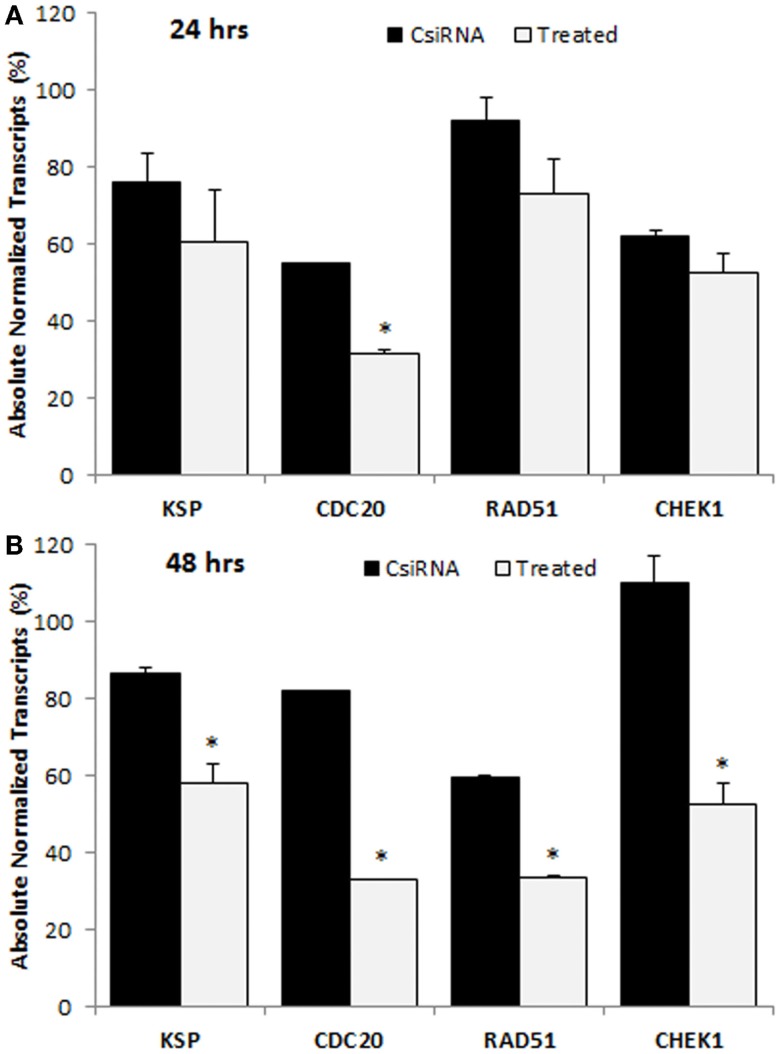
**Digital droplet PCR (ddPCR) analysis in MDA-MB-435WT cells after 24 h (A) and 48 h (B) of treatment with indicated siRNAs**. The percentage of quantity of transcripts was calculated based on the transcripts level of untreated cells (100%). The significance (*) at *p* < 0.05 was calculated for specific siRNA-treated group based on CsiRNA.

The down-regulation in the levels of mRNA transcripts of targeted proteins was analyzed with ddPCR in MDA-MB-435WT by determining absolute transcripts quantities. The levels of KSP transcripts were not significantly decreased in KSP siRNA-treated cells after 24 h of siRNA treatment (Figure 7A); however, a significant decrease was obtained after 48 h of treatment (Figure 7B). The amount of KSP transcripts in treated cells was ~60%, indicating that a relatively small change in levels of KSP transcripts inhibited cell growth drastically as KSP siRNA decreased the cell growth >70% (Figure 4). The CDC20 and RAD51 siRNAs silenced their mRNA targets more effectively compared to other cell cycle proteins as only ~30% transcripts were found in the siRNA-treated cells (Figure 7B). However, MDA-MB-435WT cells had escaped the effect of CDC20 and RAD51 siRNA treatment and survived with low copy numbers of CDC20 and RAD51 transcripts (Figure 4). A significant difference in the levels of CHEK1 transcripts was also found between scrambled siRNA and CHEK1 siRNA-treated cells after 48 h (Figure 7B). It was interesting to note that the levels of gene transcripts were variable among the chosen targets after the control siRNA treatment; while some transcripts were not affected (e.g., RAD51 at 24 h and CHEK1 at 48 h), others displayed as much as ~40% reduction in transcript levels as compared to untreated control cells (e.g., CDC20 at 24 h and RAD51 at 48 h). The reason(s) for such a variation is not known.

### DsiRNA delivery against cell cycle proteins

**Figure 8:**
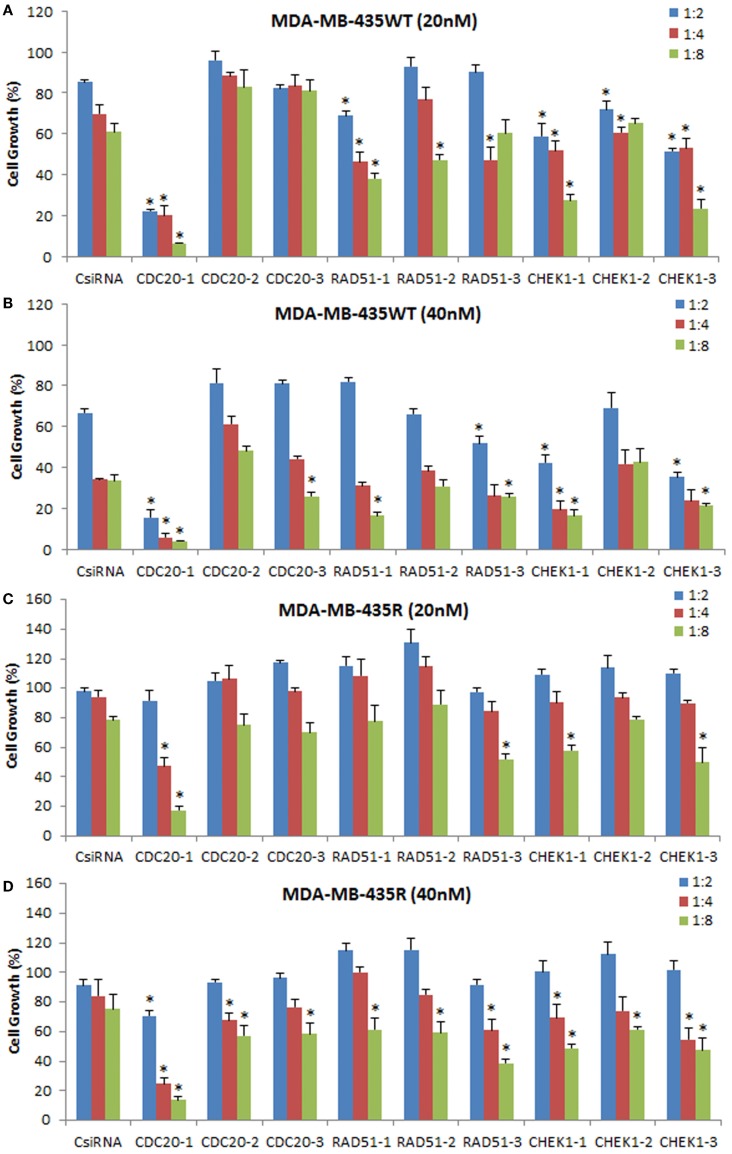
**Inhibition of cell growth using DsiRNAs against CDC20, RAD51, and CHEK1 at 20 and 40 nM DsiRNA concentrations with different DsiRNA:PEI–LA ratios in MDA-MB-435WT (A,B) and MDA-MB-435R (C,D)**. For each target proteins, three different DsiRNA isoforms were used. The significance (*) at *p* < 0.05 was calculated for specific DsiRNA treated group compared to scrambled DsiRNA (CsiRNA) at the equivalent concentration/ratio used.

To explore the effectiveness of alternative RNAi reagents, three DsiRNAs targeting different locations in mRNA was delivered against CDC20, RAD51, and CHEK1 in MDA-MB-435WT and MDA-MB-435R using PEI–LA. The siRNA concentrations were 20 and 40 nM and 1:2, 1:4, and 1:8 DsiRNA:polymer ratios were used (Figure 8). The CDC20-1 DsiRNA was the most effective among three DsiRNAs as the inhibition of cell growth was >80% at 20 and 40 nM concentrations in MDA-MB-435WT. The CDC20-2 and CDC20-3 DsiRNAs were not effective in MDA-MB-435WT cells. The DsiRNA:PEI–LA ratios 1:4 and 1:8 at 40 nM were toxic as only ~30% cell growth was found in scramble DsiRNA treated cells (Figure 8B). Similarly, a significant decrease in cell growth was observed by delivering CDC20-1 DsiRNA to MDA-MB-435R, and CDC20-2 and CDC20-3 DsiRNAs were again not as effective as CDC20-1 in this cell at 20 nM (Figure 8C) and 40 nM (Figure 8D). Again, CDC20-1 inhibited the growth of MDA-MB-231WT cells significantly, and CDC20-2 and CDC20-3 were unable to decrease the cell growth (Figure S4 in Supplementary Material). All three DsiRNAs against CDC20 were effective in MCF7 cells at 40 and 60 nM DsiRNAs (Figure S4 in Supplementary Material). However, the DsiRNA:polymer ratio 1:4 inhibited more MCF7 growth compared to 1:2 and 1:8 ratios, which was different from MDA-MB-435 cells, where the higher ratios inhibited cell growth more effectively.

The DsiRNAs against RAD51 were not as effective as CDC20-1 (Figure 8). RAD51-1 inhibited the MDA-MB-435WT cell growth ~20% compared to scrambled DsiRNA at 20 nM with 1:4 and 1:8 DsiRNA to PEI–LA ratios. No drastic decrease in the cell growth was detected by delivering RAD51-2 and RAD51-3 to MDA-MB-435WT cells at 20 and 40 nM. The significant decrease in the MDA-MB-435R cell growth was only observed at 40 nM of DsiRNA at 1:8 ratio with all three DsiRNAs (Figure 8D). RAD51 DsiRNAs were not effective in MDA-MB-231WT cells (Figure S4 in Supplementary Material). However, MCF7 cells were more sensitive to all three RAD51 DsiRNAs, which inhibited cell growth drastically at 60 nM of DsiRNA (Figure S4 in Supplementary Material).

All CHEK1 DsiRNAs decreased the MDA-MB-435WT cell growth significantly compared to scrambled DsiRNA at 20 nM with various ratios (Figure 8A). However, 1:8 DsiRNA:PEI-LA ratio at 20 nM was the most effective ratio in MDA-MB-435WT as ~40% cell growth was inhibited. Since the higher ratios at 40 nM of DsiRNA were toxic, the inhibition of cell growth by CHEK1 DsiRNAs alone was quite low at 40 nM (Figure 8B). Only the higher CHEK1 DsiRNA:polymer ratios inhibited the MDA-MB-435R cells significantly at 20 and 40 nM of DsiRNAs (Figure 8D). The CHEK1 DsiRNAs were not effective in MDA-MB-231WT cells (Figure S4 in Supplementary Material). CHEK1-1 decreased the MCF7 cell growth drastically at 40 and 60 nM of DsiRNA at different ratios. CHEK1-2 and CHEK1-3 DsiRNAs failed to inhibit the cell growth drastically in MCF7 cells (Figure S4 in Supplementary Material).

The sensitizing effect of DsiRNAs for doxorubicin was not observed in MDA-MB-435R at 20 and 40 nM, as a similar inhibition of cell growth was observed between DsiRNA/doxorubicin-treated and DsiRNA treated cells (data not shown).

### *In vivo* CDC20 DsiRNA therapy

**Figure 9:**
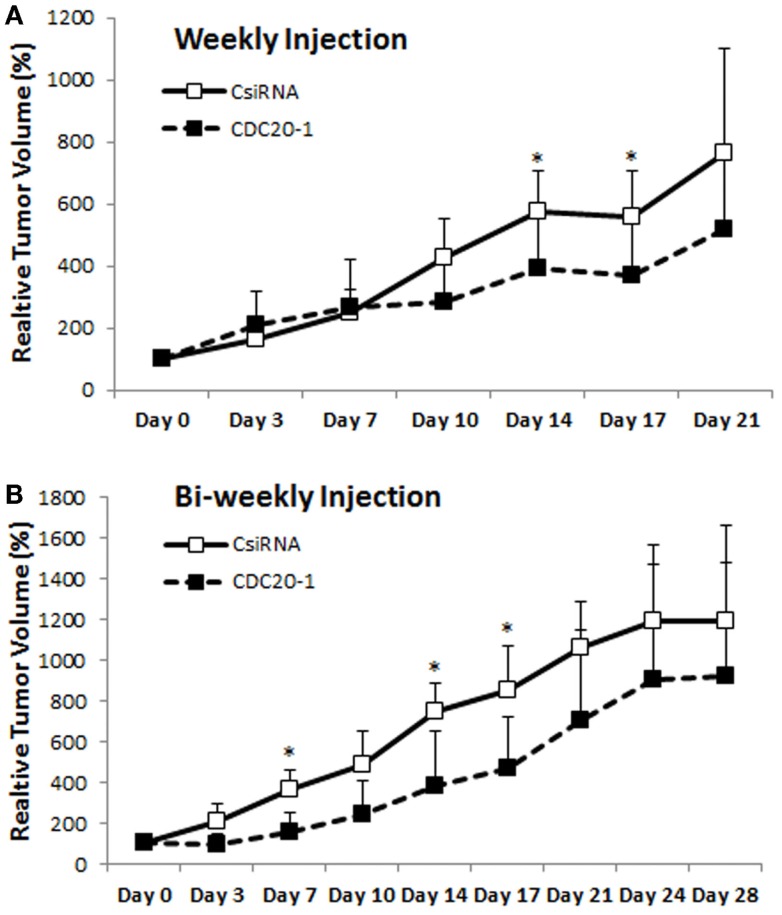
**Effect of CDC20 DsiRNA treatment *in vivo***. Xenografts of MDA-MB-435WT were established in nude mice, and were treated with a scrambled DsiRNA (CsiRNA) and CDC20-1 DsiRNA. Relative tumor volume for weekly injection [**(A)**; *n* = 6 and *n* = 5 in CsiRNA and CDC20-1 groups, respectively] and bi-weekly injection [**(B)**; *n* = 3 and *n* = 4 in CsiRNA and CDC20-1, respectively] groups, are summarized (only positive SDs are shown for clarity). The time points that showed a significant decrease in the volume of CDC20-1 DsiRNA treated tumors compared to CsiRNA treated tumor are indicated with an asterisk (*p* < 0.05).

Since the CDC20-1 DsiRNA led to >80% growth inhibition in MDA-MB-435WT cells *in vitro* (more so than the CDC20 siRNA from library screens), we further evaluated its efficacy *in vivo* by injecting DsiRNA/PEI-LA complexes to breast cancer xenografts weekly and bi-weekly subcutaneously in the vicinity of tumor. In the weekly injection group, the initial growth of scrambled and CDC20-1 DsiRNA treated tumor was similar (Figure 9A). However, the growth of tumor was suppressed after the second injection of CDC20-1 DsiRNA and a significant difference compared to scrambled DsiRNA treated tumor was achieved on day 14. Similarly, the third injection also decreased the growth of CDC20-1 DsiRNA treated tumor significantly on day 17. In the bi-weekly injection groups, the slower growth was evident with CDC20-1 DsiRNA treated group from the beginning of the study, where the differences between the CDC20-1 and scrambled DsiRNA were significant on day 7 and 14 (Figure 9B). The tumor growth was retarded significantly after the second injection of CDC20-1 DsiRNA on day 17 and the difference in growth rate between scrambled and CDC20-1 DsiRNA treated tumor started decreasing gradually thereafter.

## Discussion

The siRNA mediated RNAi has become a powerful tool for its specificity and efficiency to knock-down targets that cannot be readily down-regulated by conventional chemotherapy (McManus and Sharp, 2002; Kim and Rossi, 2007). However, an efficient delivery system has to be developed for a functional siRNA effect (Pecot et al., 2011; Bora et al., 2012). Here, we report polymeric delivery systems, PEI-LA and PEI-CA, for siRNA therapy against cell cycle proteins in breast cancer cells. Lipid moieties that have been used to substitute amines of PEI were speculated to increase the interaction of anionic cell membrane with complexes (nanoparticles) of siRNA formed with cationic polymers, which in turn facilitate the entry of anionic siRNA into the cell. The optimal ratio of polymer to siRNA for each cell-line needs to be determined as a balance between the cytotoxicity of the polymer (lower cytotoxicity at lower ratios) and effective siRNA delivery (increased siRNA delivery at higher ratios). The current study mostly utilized *in vitro* cell models since, at the onset of study, little was known about the feasibility of silencing the newly explored targets to obtain a therapeutic effect. Detailed studies on dose–response relationships, relative potency of silencing each identified target, and details of siRNA delivery system (efficiency and undesired cytotoxicity) were thoroughly explored *in vitro*. With the critical insight generated in this study, further *in vivo* studies are warranted to better explore the potential of the identified targets.

The arrest of cell cycle by knocking out or inhibiting specific proteins was explored previously by others (Schwartz and Shah, 2005; Satyanarayana and Kaldis, 2009). Our results (based on PCR analysis and inhibition of cell growth) highlighted three specific mediators, namely CDC20, RAD51, and CHEK1, as therapeutic targets in breast cancer cells. Western blot analysis to assess protein levels as a result of specific siRNA delivery would have been additionally useful to better validate these targets, but the inhibition of cell growth by specific siRNAs was considered a strong indication for their importance and a practical end-point to identify leads. The CDC20 activates the anaphase-promoting complex (APC) in the cell cycle, which initiates chromatid separation and entrance into anaphase (Weinstein, 1997). RAD51 repairs the DNA double-strand break during homologous recombination (Galkin et al., 2006). CHEK1 has kinase activity and phosphorylates CDC25, an important phosphatase for entry of the cell into mitosis (Chen et al., 2003). There are already a precedent for the roles of unregulated CDC20, RAD51, and CHEK1 in cancer development and progression. CDC20 has been found to be overexpressed in many cancer types (Takahashi et al., 1999; Kim et al., 2005b; Iacomino et al., 2006; Ouellet et al., 2006; Kidokoro et al., 2008), which may deregulate activation process of APC and often result in multinucleation, premature anaphase promotion, and mis-segregation of chromosomes, and leads to chromosomal instability and defect in spindle assembly checkpoint response (Mondal et al., 2007; Wang et al., 2013). Given the role of RAD51 in DNA double-strand break repair (Galkin et al., 2006), RAD51 up-regulation increases the number of recombination events that may lead to defective DNA strands (Richardson et al., 2004). In addition, spontaneous recombination frequency may increase in mammalian cells because of overexpression of RAD51, which ultimately provides resistance to chemotherapy (Vispé et al., 1998; Klein, 2008). CHEK1, on the other hand, is an essential cell cycle protein to maintain genomic stability. Syljuåsen et al. (2005) suggested that CHEK1 is a required protein to avoid uncontrolled increase in DNA replication, thereby protecting against DNA breakage. Although this literature supported all three targets for RNAi based cancer therapy, only a few studies attempted to silence CDC20, RAD51, and CHEK1 expression by siRNA (Syljuåsen et al., 2005; Taniguchi et al., 2008; Tsai et al., 2010). Commercial carriers such as RNAiFect™ reagent (Qiagen), Lipofectamine™ 2000 and Oligofectamine™(Invitrogen) were used to deliver CDC20, RAD51, and CHEK1 siRNA, respectively, and these studies were conducted in pancreatic, non-small-cell lung carcinoma (NSCLC), and osteosarcoma cell-lines. The breast cancer therapy investigated here might be an additional indication for these targets in RNAi therapy.

Our studies indicated that MDA-MB-435R cells were more responsive to siRNA treatment as compared to MDA-MB-231R. One possible reason might be that the polymeric carrier has not delivered siRNAs effectively to MDA-MB-231R cells. We previously reported that MDA-MB-231R cells displayed lower uptake compared to MDA-MB-435R cells under identical culture conditions (Aliabadi et al., 2013b), so that lower quantitative delivery of intracellular siRNA could be one of the reasons for lower efficacy in these cells. Another possibility is that the targeted cell cycle proteins may not be as crucial for the survival of MDA-MB-231R, unlike the MDA-MB-435R cells, and the MDA-MB-231R cells may have circumvented the effect of siRNA treatment by recruiting alternative mediators. However, individually prepared siRNAs against three cell cycle proteins showed lower efficacy in MDA-MB-435WT (Figures 4 and 7) and MDA-MB-435R (Figure 6), and these siRNAs were not effective at all in MDA-MB-231WT, MDA-MB-231R, and MCF7 cells (Figures S2 and S3 in Supplementary Material), which might be an indication of these siRNAs not being efficiently incorporated into the RISC assembly. In order to address this possibility, we determined the efficacy of DsiRNAs against the three cell cycle proteins. DsiRNA (27 base pairs) interacts with the dicer enzyme before its incorporation into RISC assembly, leading to increased potency by engaging to the natural siRNA processing pathway (Kim et al., 2005a). Three 27 bp DsiRNAs for each target were not uniformly effective than the 21 bp siRNA used in this study, but we are cognizant of the fact that different regions of mRNA were targeted with each RNAi reagent and this might have contributed to variation in their efficacy. However, the CDC20-1 DsiRNA was clearly the most effective among the tested reagents, which led us to determine its efficacy in a xenograft model. The CDC20-1 DsiRNA was able to decrease the tumor growth in both weekly and bi-weekly injection groups. The retardation of tumor growth with CDC20-1 DsiRNA was not as robust as other studies in the literature. However, the DsiRNA dose used here was 2 μg (~0.08 mg/kg/day), which was quite low compared to 4–10 μg of siRNA used in intratumoral injections in previous studies and some higher doses (up to 40 μg of siRNA) used in other modes of administrations (Behlke, 2006). We did not employ intratumoral injections since that might alter tumor growth patterns and complicate the interpretation of tumor growth data. Moreover, we reduced the number of injections in our study to weekly and bi-weekly, leading to a large interval between the injections as 1–5-day durations were frequently employed in previous *in vivo* studies (Behlke, 2006). Even though the dose of DsiRNA and frequency of injections were low, the CDC20-1 DsiRNA was effective to slow down the growth of tumor compared to scrambled DsiRNA (Figure 9). We must, however, note that no buffer injection group was employed in the animal study, so that we could not evaluate if the scrambled DsiRNA complexes had any effect on tumor growth due to non-specific toxicity.

The resistance to the chemotherapy arises due to molecular (protein) changes in cancer cells (Luqmani, 2005). If a protein associated with drug resistance was to be down-regulated by siRNA therapy, cells could be sensitized to chemotherapy. This issue was explored in several experiments with cell cycle proteins in this study, where the silencing of particular proteins were first attempted to investigate subsequent drug (doxorubicin) response. Since doxorubicin action involves DNA intercalation to inhibit DNA replication and ultimately cell cycle arrest, we initially reasoned that protein controlling the cell cycle could be altered in doxorubicin-treated cells, as observed in MCF7 cells (AbuHammad and Zihlif, 2013). The drug-resistant MDA-MB-231R and MDA-MB-435R cells were not sensitized to doxorubicin after siRNA therapy (either as a single or dual siRNA delivery), indicating that the targeted cell cycle proteins may not be contributing to resistance against doxorubicin in breast cancer cells. Our previous studies with siRNA delivery were able to sensitize breast cancer cells by targeting anti-apoptotic proteins survivin (Montazeri Aliabadi et al., 2011) and Mcl-1 (Aliabadi et al., 2013a), so that this class of proteins (rather than cell cycle proteins) might be more suitable to target for chemo-sensitization. However, beyond chemo-sensitization, cell cycle proteins could serve as targets to inhibit metastasis, since delivering specific siRNAs against survivin and cyclin B1 (with linear PEI) were found to be effective to prevent lung metastasis in a mammary adenocarcinoma model in mice (Bonnet et al., 2013).

Another cell cycle protein, KSP, has been investigated as a target for RNAi therapy and currently it is being evaluated at clinics (Tabernero et al., 2013). As KSP is a microtubule-based motor protein and plays a critical role during mitosis to separate centrosome and to assemble bipolar spindle, knock-down of KSP expression leads to cell cycle arrest, and ultimately to cell death. KSP was another effective target with the described polymeric delivery system. We previously observed that silencing multiple targets by delivering multiple siRNAs simultaneously led to improved therapeutic responses (Aliabadi et al., 2013a,b); however, this was not the case here when KSP was combined with siRNAs targeting one of the cell cycle proteins. KSP on its own seemed to be effective enough to eradicate >70% cells. KSP with vascular endothelial growth factor (VEGF) siRNA is in clinical use (Tabernero et al., 2013), so that other targets beyond the cell cycle proteins might still be suitable for combinational therapy with KSP siRNA. Lipid nanoparticles (LNP) are used to deliver KSP–VEGF siRNAs intravenously, which form micelles around siRNA to protect its extracellular degradation. The modified PEI used for delivery of KSP siRNA here interacts electrostatically with siRNA and might provide an alternative delivery system for this clinically useful siRNA.

In conclusion, we report effective polymers derived from lipid-substituted 2 kDa PEI to target proteins involved in cell cycle regulation in breast cancer cells. No clear difference was evident in our study whether a CA or LA modification of PEI was more effective. The proteins CDC20, RAD51, and CHEK1 were identified as promising targets among the cell cycle proteins for non-viral RNAi therapy. The specific type of RNAi reagent, siRNA (21 bp), or DsiRNA (27 bp), was found to influence the efficacy of therapy for individual targets, but more studies are needed to clarify the exact reason for the differences. Although we expected the siRNA therapy against cell cycle proteins to sensitize the cells with chemotherapy, no such effect was evident when doxorubicin was employed as a sensitizing drug. Nevertheless, a DsiRNA against CDC20 was the most potent RNAi reagent in our hands, and it also effectively slowed the growth of breast cancer xenografts in an animal model. The present study highlighted the importance of cell cycle protein targets in breast cancer therapy, and demonstrated an effective delivery system for down-regulation of cell cycle proteins.

## Conflict of Interest Statement

The authors declare that the research was conducted in the absence of any commercial or financial relationships that could be construed as a potential conflict of interest.

## Supplementary Material

The Supplementary Material for this article can be found online at http://www.frontiersin.org/Journal/10.3389/fbioe.2015.00014/abstract
