# Supplementary material for: Targeting Cell Cycle Proteins in Breast Cancer Cells with siRNA by Using Lipid-Substituted Polyethylenimines
Source: Front Bioeng Biotechnol. 2015 Feb 16;3:14. doi: 10.3389/fbioe.2015.00014 (PMC4329877; doi:10.3389/fbioe.2015.00014)
Supplement: Supplementary file 1 [file Presentation_1.PDF]

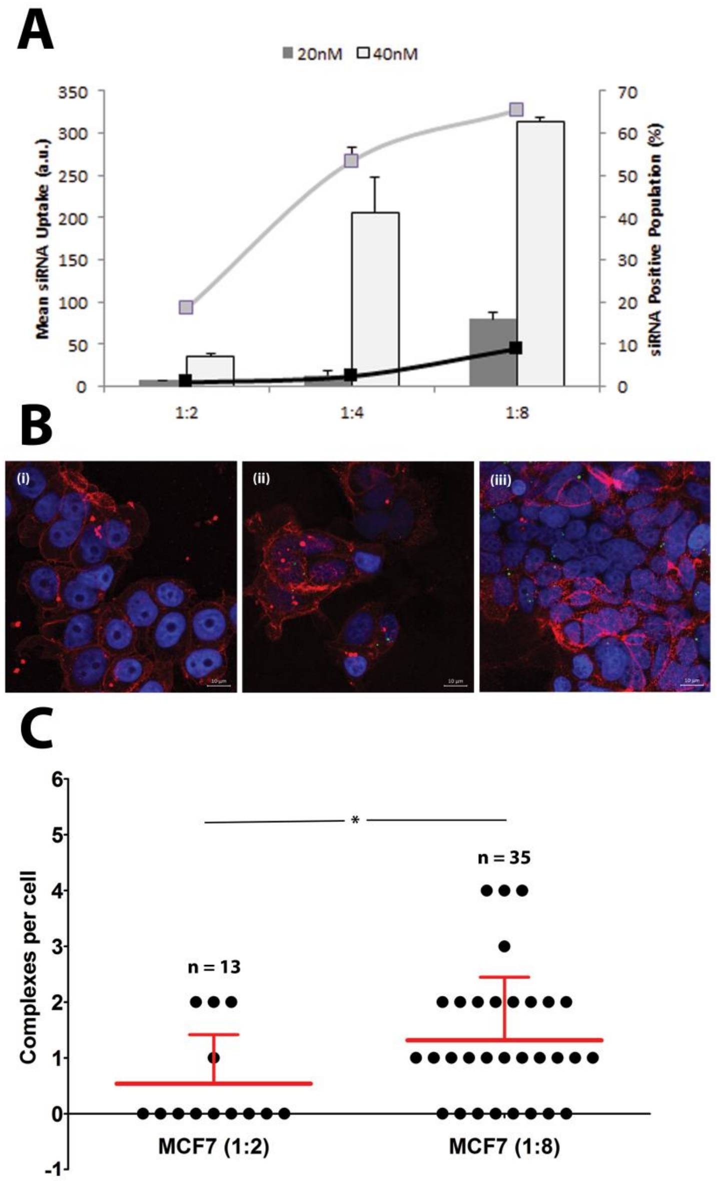

Figure 1S. (A) Uptake of FAM-labeled siRNA complexes by flow cytometry at 20 nM and 40 nM siRNA using 1:2, 1:4 and 1:8 siRNA:PEI-LA ratios after 24 hrs treatment in MCF7 cells. The results are summarized as mean FAM-siRNA uptake (bars) and as percentage of FAM-siRNA positive cell population (lines). **(B)** Confocal microscopy to determine the uptake of FAM-labeled siRNA complexes at 40 nM siRNA with 1:2 (ii) and 1:8 (iii) siRNA:PEI-LA ratios after 24 hrs treatment. Purple, red and green colors represent nuclei, cytoplasm and siRNA complexes, respectively. Non-labeled scrambled siRNA was transfected as a control (i). **(C)** The number of visible complexes per cell (as quantitated from confocal microscopy images) at 1:2 and 1:8 siRNA:PEI-LA ratios. The uptake was significantly different between 1:2 and 1:8 ratios (* at $p<0.05$).

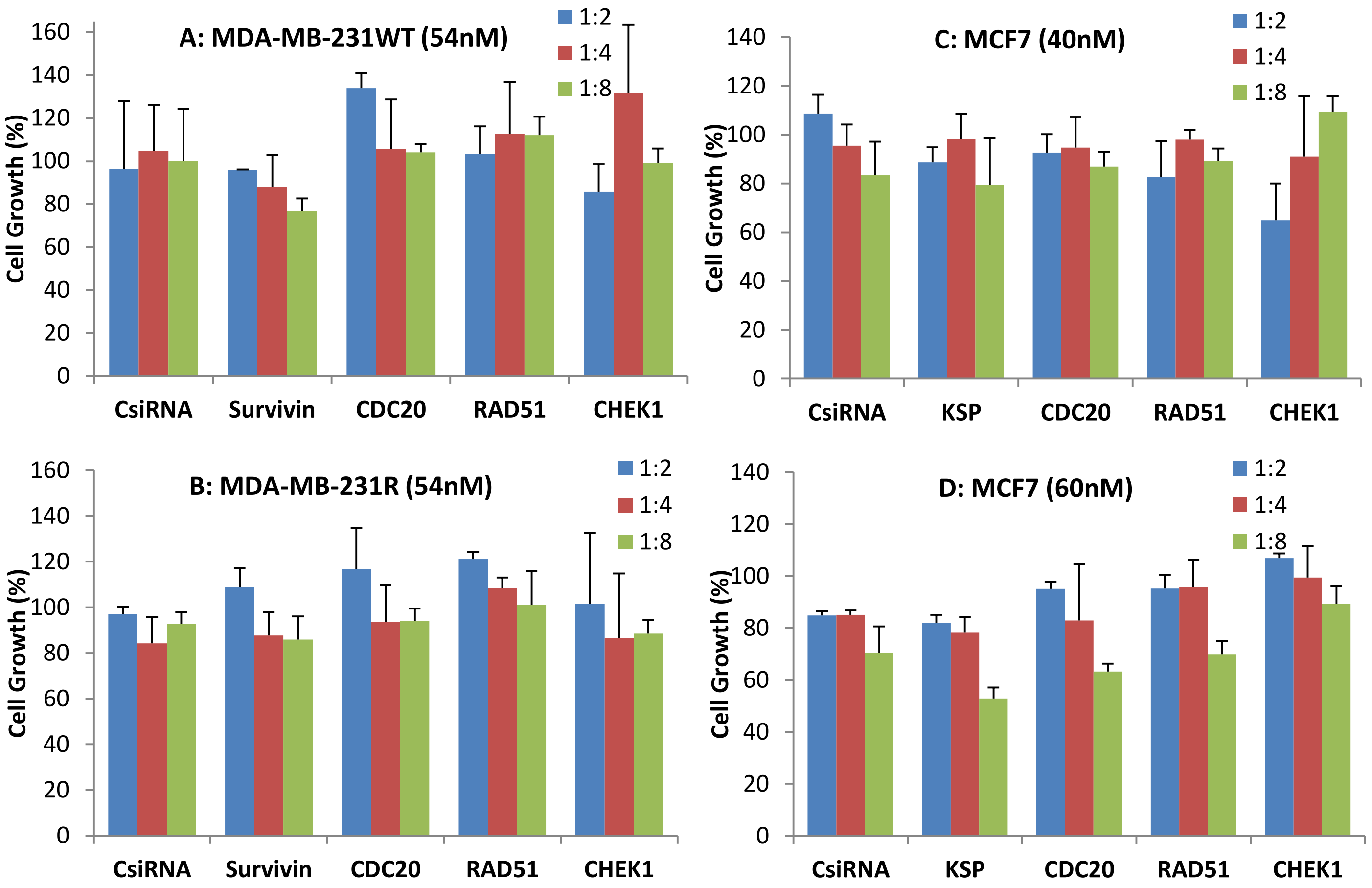

Figure 2S. The effects of cell cycle protein specific siRNAs in MDA-MB-231 (wild type, WT and multidrug resistant, R) at 54 nM and in MCF7 cells at 40 nM and 60 nM siRNA. In addition to KSP, CDC20, RAD51 and CHEK1 specific siRNAs, a specific siRNA against the anti-apoptosis protein, survivin was delivered using PEI-LA at 1:2, 1:4 and 1:8 siRNA:polymer ratios. The inhibition of cell growth by MTT assay indicated that the specific siRNA treatments were not effective in MDA-MB-231 and MCF7 compared to scrambled siRNA (CsiRNA).

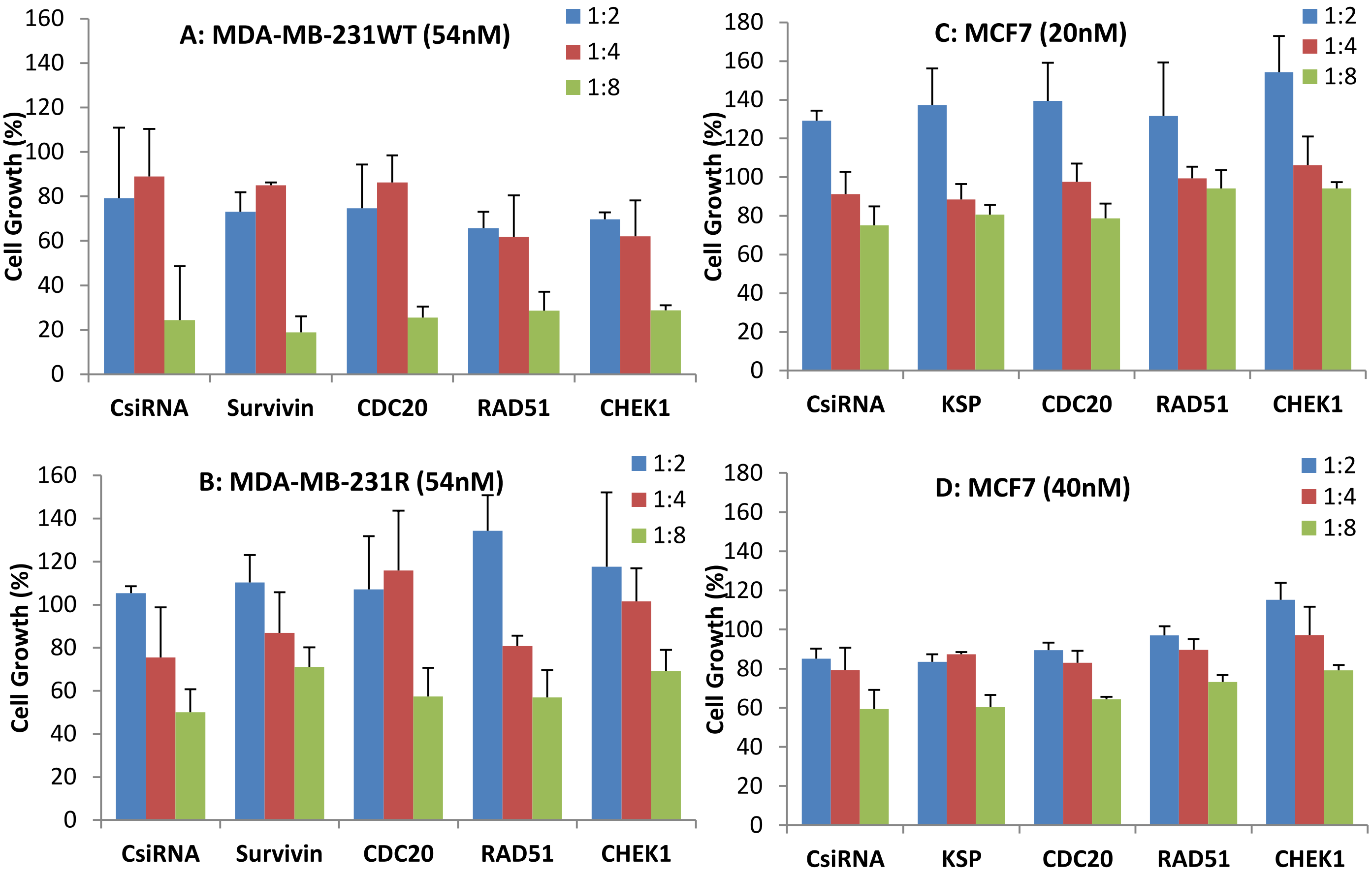

Figure 3S. The cell cycle proteins, KSP, CDC20, RAD51 and CHEK1 with survivin were validated using PEI-CA in MDA-MB-231 (wild type, WT and multidrug resistant, R) at 54 nM siRNA, and in MCF7 at 20 nM and 40 nM siRNA concentrations. The results of the inhibition of cell growth assay by MTT indicated that the siRNA treatments were not effective in MDA-MB-231 and MCF7 compared to scrambled siRNA (CsiRNA).

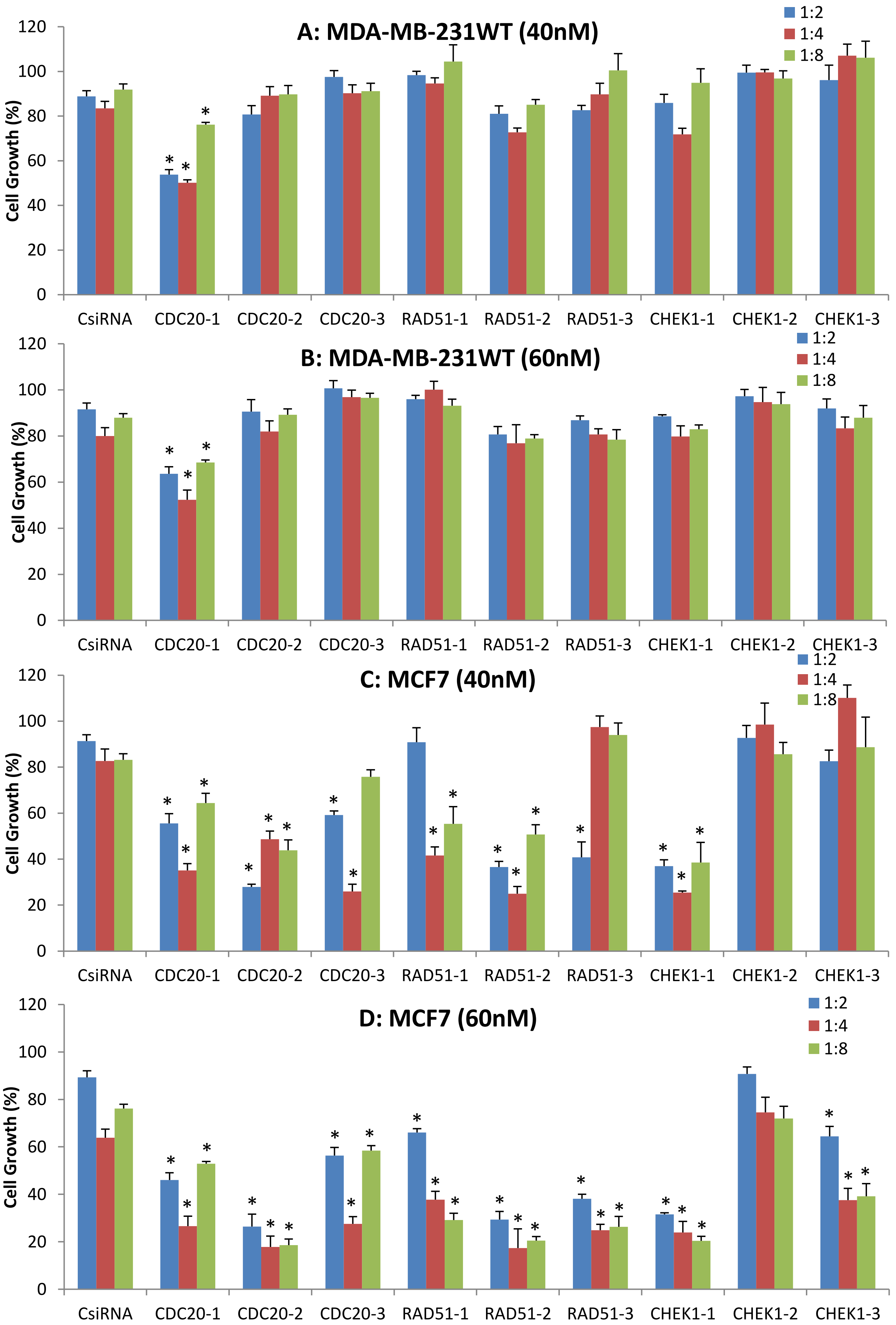

A: MDA-MB-231WT (40nM)
B: MDA-MB-231WT (60nM)
C: MCF7 (40nM)
D: MCF7 (60nM)
Cell Growth (%)
1:2
1:4
1:8
0
20
40
60
80
100
120
CsiRNA
CDC20-1
CDC20-2
CDC20-3
RAD51-1
RAD51-2
RAD51-3
CHEK1-1
CHEK1-2
CHEK1-3

Figure 4S. Inhibition of cell growth using DsiRNAs against CDC20, RAD51 and CHEK1 at 40 nM and 60 nM DsiRNA concentrations with different DsiRNA:PEI-LA ratios in MDA-MB-231WT and MCF7. For each target proteins, three different DsiRNA isoforms were used. The significance (*) at $p<0.05$ was calculated for specific DsiRNA treated group based on scrambled DsiRNA (CsiRNA).
